# Teprotumumab for thyroid eye disease: early response is not required for benefit

**DOI:** 10.1038/s41433-021-01539-5

**Published:** 2021-06-28

**Authors:** Shoaib Ugradar, Yao Wang, Tunde Mester, George J. Kahaly, Raymond S. Douglas

**Affiliations:** 1 The Jules Stein Eye Institute University of California, Los Angeles, Los Angeles, USA; 2 Cedars-Sinai Medical Center, Los Angeles, CA USA; 3 Department of Medicine I, Johannes Gutenberg University (JGU) Medical Center, Mainz, Germany

**Keywords:** Outcomes research, Thyroid diseases

## Abstract

**Purpose:**

In recent trials, 50% of patients treated with teprotumumab for thyroid eye disease had significant improvements in proptosis at 6 weeks. However, a small subgroup of patients did not have a significant response by week 12. We examine the outcomes at week 24 in patients from both trials who had little or no proptosis response at week 12.

**Design:**

In this post hoc analysis, data from teprotumumab-treated patients in the placebo-controlled randomized phases 2 and 3 trials were reviewed.

**Methods:**

Patients treated with teprotumumab or placebo with a ≤2 mm reduction from baseline in proptosis at week 12 and completed assessments at both the weeks 12 and 24 visits were included. The main outcome measures were a change in proptosis, clinical activity score (CAS) and diplopia in response to teprotumumab therapy at baseline and weeks 6, 12, 18, and 24.

**Results:**

From the phases 2 and 3 studies, 24 patients from the treated and placebo groups were included for analysis (48 total). In the teprotumumab group, of the 24 who had no improvement in proptosis (≥2 mm from baseline) at 12 weeks, 15 (63%) demonstrated a clinically significant improvement at week 24. No patients from the 24 placebo patients had a clinically significant improvement in proptosis at 12 weeks, and 24 weeks. At week 12, 22 patients (92%) in the teprotumumab group had a significant reduction in the CAS (≥2 points) and at 24 weeks all patients achieved this reduction. At week 12, 11 (46%) patients from the placebo group had a significant improvement, while 10 (42%) had a significant improvement at 24 weeks. 22 of the 24 patients (92%) in the teprotumumab group had a diplopia grade > 0 at baseline. At week 12, 12 of the 22 (55%) had improvement in diplopia ≥ 1 grade. By week 24, 16 patients (73%) had an improvement in diplopia ≥ 1 grade. In the placebo group, 15 (63%) had significant diplopia. At week 12, 3 (20%) from this group had improvement in diplopia ≥ 1 grade, while at 24 weeks this number rose to 4 (27%).

**Conclusions:**

There is variability in the time taken to manifest a clinically significant response to teprotumumab, some patients my need a longer time to respond.

## Introduction

Thyroid eye disease (TED) is a complex autoimmune condition that results in disfiguring orbital changes that may be associated with the development of diplopia and optic neuropathy [1]. The disease begins with acute inflammation of the orbital and periorbital soft tissues, during which symptoms may include dry eyes, orbital pain, irritation, and eyelid retraction. Disfiguring and debilitating proptosis, diplopia, and strabismus also occur. Over time, the disease becomes chronic where inflammation decreases, but damage to the orbital tissues may become permanent. Immunomodulation using steroids [2], rituximab [3], or tocilizumab [4] may be effective in controlling the initial inflammatory signs and symptoms of TED, but none of these treatments have been found to significantly improve proptosis and diplopia in controlled trials [5, 6]. The changes to appearance and vision impact patients’ self-confidence and ability to carry out daily tasks, and thus their quality of life [7].

TED develops in 40–50% of patients who have a diagnosis of Graves’ disease (GD). Proptosis and misalignment of the eyes result from inflammation and expansion of the retroorbital fat and muscle. Overexpression of the insulin-like growth factor 1 receptor (IGF-1R) and its interaction with the thyrotropin receptor (TSH-R) is a key pathogenic feature of this disease [8]. The TSH-R and IGF-1R form a physical and functional complex on the cell membrane of fibroblasts, B cells, and T cells [9]. Autoantibodies to IGF-1R bind to the complex, leading to increased production of hyaluronan, proinflammatory cytokines [10], IL-2 [11], TNF-α [12], and IL-8 [13] by T cells and monocytes, and the expansion of adipose tissue.

Teprotumumab is a fully human monoclonal immunoglobulin G1 to IGF-1R that was recently approved for the treatment of TED in the USA [14]. Binding of teprotumumab inhibits signaling through the IGF-R1/TSH-R complex and the downstream pathways. In phases 2 and 3, randomized, double-masked clinical trials (NCT01868997 and NCT03298867) [15, 16] undertaken in patients with active TED, teprotumumab was more effective than placebo in reducing proptosis, diplopia, and inflammation [15]. In approximately one half of treated patients, the impact of teprotumumab was rapid, with significant improvements in proptosis, diplopia, and clinical activity score (CAS) observed at 6 weeks [15]. While the majority of patients in the trials demonstrated a rapid response and sustained improvement in proptosis during the phases 2 and 3 trials, a small subgroup of patients did not have a clinically meaningful (≥2 mm) reduction in proptosis at week 12. This has led some to speculate that if early response is not seen the drug can be discontinued. Here we examine the outcomes at week 24 in patients from both trials who had little or no proptosis response at week 12.

## Methods

The phases 2 and 3 clinical studies adhered to the tenets of the Declaration of Helsinki were performed in accordance with the Health Insurance Portability and Accountability Act and were approved by the sites’ institutional review boards. All patients provided written consent for the studies.

### Patients and studies

This post hoc analysis included data from teprotumumab-treated patients in the phases 2 and 3 trials (NCT01868997 and NCT03298867) along with age and sex matched controls, which have been reported elsewhere [12, 13]. Patients and controls were included in this analysis if they had less than 2 mm reduction (which was the primary outcome of interest) from baseline in proptosis at week 12, and completed assessments at both the weeks 12 and 24 visits. Patients received eight infusions of teprotumumab (10 mg/kg for the first infusion and 20 mg/kg for subsequent infusions) every 3 weeks over 24 weeks [15, 16].

### Measurement of clinical outcomes

Observed data on clinical measurements from both the phases 2 [16] and 3 [15] trials were included in the present analysis. The outcome measures have been reported previously and are described briefly here. The more severely affected (proptotic) eye was designated as the study eye. Proptosis was assessed using a Hertel exophthalmometer. A reduction of ≥2 mm was considered clinically significant. Changes in diplopia grade were assessed using the Gorman subjective diplopia score [17] (range 0–3). A score of 0 indicates no diplopia; 1, intermittent diplopia; 2, inconstant diplopia; and 3, constant diplopia. An improvement ≥1 grade is considered as clinically significant. Inflammation was assessed using the seven-point CAS [18], which scores the presence of each of the following signs: retrobulbar eye pain, pain on eye movement, eyelid erythema, eyelid swelling, conjunctival redness, chemosis, inflammation of the caruncle or plica. A CAS score ≤1 is indicative of disease inactivation [18]. All measurements were conducted at baseline and weeks 6, 12, 18, and 24.

### Statistical analysis

Statistical analysis was performed using SPSS version 22.0 (SPSS, Inc., Chicago, Illinois, USA). The difference between proptosis measurements at various time points (comparison of two time points) was calculated using a dependent *t*-test, while the difference between CAS and diplopia scores at different time points was analyzed using the Wilcoxon Signed Rank test. A Pearson correlation was used to review associations between age and proptosis, while a Spearman’s rank correlation coefficient was calculated to review associations between age, duration of disease, and CAS. Statistical significance was defined as *p* < 0.05.

## Results

A total of 84 patients were randomized to teprotumumab across the phases 2 [15] and 3 [16] studies.

### Teprotumumab group

Twenty-four patients (10 males, 14 females), with proptosis reduction from baseline <2 mm at week 12, who received teprotumumab were included in the present study. Eight patients were from the phase 3 study and 16 were from the phase 2 study. The mean (SD) age was 48.5 (11.9) years. The demographic data of patients included in the study are shown in Table 1. The mean (SD) duration of diagnosed TED was 6.1 (2.2) months.

**Table 1:** Demographics and disease characteristics at baseline of patients included in the study.

	Teprotumumab (*n* = 24)	Placebo (*n* = 24)
Age (years), mean (SD)	48.5 (11.9)	50.29 (11.51)
Gender, *n*		
Male	10	10
Female	14	14
Race, *n*		
White	21	20
Black	1	1
Asian	1	1
Native Hawaiian/Pacific Islander	1	2
Years since diagnosis of Graves’ disease, mean (SD)	2.6 (5.7)	2.1 (2.5)
Months since diagnosis of thyroid eye disease, mean (SD)	6.1 (2.2)	6.4 (2.71)
Tobacco use history, *n*		
Nonsmoker	18	20
Smoker	6	4
Proptosis, mean (SD), mm	22.2 (3.2)	22.7 (3.5)
CAS (seven-point scale), Mean (SD)	5.1 (0.9)	5.5 (1.1)
Patients with diplopia, *n*	22	15
Intermittent	9	6
Inconstant	6	3
Constant	7	6

*CAS* clinical activity score, *SD* standard deviation.

### Placebo group

Twenty-four patients (10 males, 14 females), with proptosis reduction from baseline <2 mm at week 12, who received placebo were included in the present study. Nineteen patients were from the phase 3 study and 5 were from the phase 2 study. The mean (SD) age was 50.3 (11.5) years. The demographic data of patients included in the study are shown in Table 1. The mean (SD) duration of diagnosed TED was 6.4 (2.7) months.

### Clinical outcomes at 12 and 24 weeks

#### Proptosis

**Fig. 1:**
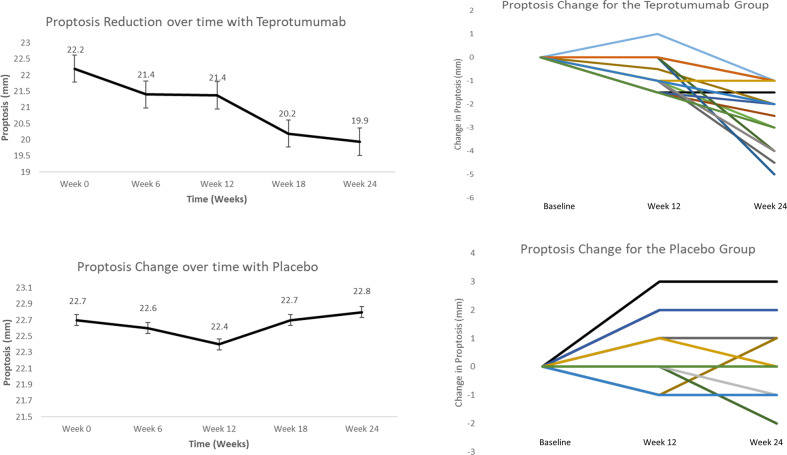
Mean reduction from baseline in proptosis over time with teprotumumab and placebo in patients with proptosis reduction < 2 mm at week 12. Bars indicate Standard Error (SE). 1b: Reduction in proptosis over time for individual patients.

**Fig. 2:**
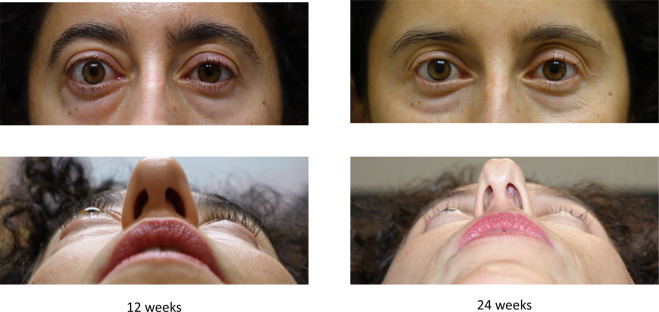
Clinical photograph of a patient with TED, treated with teprotumumab at 12 weeks and 24 weeks.

Of the 24 patients who did not have a clinically significant reduction in proptosis (<2 mm) at 12 weeks, 15 (63%) demonstrated a clinically significant improvement (≥2 mm from baseline) at week 24. At 24 weeks, mean (SD) proptosis reduction from baseline was −2.3 (1.3) mm, which is clinically significant (Fig. 1). There was a greater reduction in proptosis following the second half of the treatment course (−1.5 mm) compared to the first half (−0.8 mm). The difference in proptosis reduction between responders and nonresponders at week 24 was not related to age (*p* = 0.6), duration of disease (*p* = 0.8), or sex (*p* = 0.4). From the placebo group, of the 24 patients who did not have a clinically significant reduction in proptosis (<2 mm) at 12 weeks, 0 demonstrated a clinically significant improvement (≥2 mm from baseline) at week 24. At 24 weeks, mean (SD) proptosis increased from baseline by 0.1 (1.1) mm (Figs. 1 and 2).

#### Clinical activity score

At week 12, 22 patients (92%) in the teprotumumab group had a clinically significant reduction in the CAS (≥2 points) and at 24 weeks all of the patients achieved this reduction. The mean (SD) of CAS was 5.1 (0.9) at baseline, 2.3 (1.5) at week 12 and 1.3 (1.2) at week 24 (Fig. 2). Overall, after 24 weeks, there was a mean (SD) reduction of 3.8 (1.0) in the CAS from baseline, which was significant (*p* < 0.01). At baseline, all 24 patients had CAS ≥ 4. At Week 12, 7 of the 24 patients (29%) had a CAS ≤ 1 and at week 24, 15 patients (63%) had CAS ≤ 1.

**Fig. 3:**
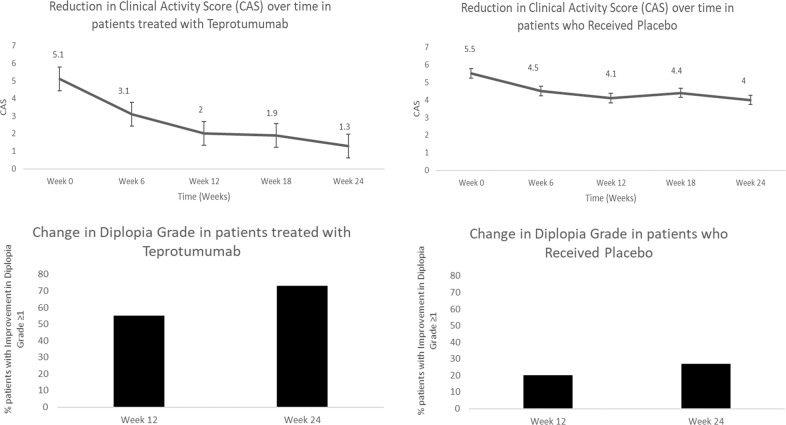
Mean reduction from baseline in CAS over time (bars indicate Standard Error (SE)) and percentage of patients with improvement in diplopia ≥ 1 grade from baseline.

From the placebo group, at week 12, 11 patients (46%) had a clinically significant reduction in the CAS (≥2 points) and at 24 weeks 10 patients achieved this reduction. The mean (SD) of CAS was 5.5 (1.1) at baseline, 4.1 (1.5) at week 12, and 4 (1.4) at week 24 (Fig. 2). Overall, after 24 weeks, there was a mean (SD) reduction of 1.4 (1.8) in the CAS from baseline, which was significant (*p* < 0.01) (Fig. 3).

#### Diplopia

Twenty-two of the 24 patients (92%) treated with teprotumumab had a diplopia grade > 0 at baseline. At week 12, 12 of the 22 (55%) had an improvement in diplopia ≥ 1 grade. By week 24, 16 patients (73%) had an improvement in diplopia ≥ 1 grade (Fig. 2). In the 24 patients, the mean (SD) Gorman diplopia score was 1.8 (1.0) at baseline and 1.1 (1.2) at week 12 (mean reduction of 0.6 (1.0)). At 24 weeks, the mean (SD) diplopia score was 0.8 (1.2), with a mean (SD) difference of −0.9 (1.1) from baseline (*p* < 0.01).

Fifteen of the 24 patients (63%) in the placebo group had a diplopia grade > 0 at baseline. At week 12, three from this group (20%) had an improvement in diplopia ≥ 1 grade. By week 24, 4 patients (27%) had an improvement in diplopia ≥ 1 grade (Fig. 2). In the 24 patients from the placebo group, the mean (SD) Gorman diplopia score was 1.3 (1.2) at baseline and 1.5 (1.2) at week 12 (Mean (SD) increase of 0.3 (0.9)). At 24 weeks, the mean (SD) diplopia score was 1.4 (1.2), with a mean (SD) difference of 0.2 (1) from baseline (*p* = 0.4) (Fig. 3).

## Discussion

Proptosis and diplopia are two sequelae of TED that have been historically difficult to treat. In combined data from two clinical trials, teprotumumab demonstrated a significant improvement in these outcomes by week 24 (the prespecified outcome timeframe) versus placebo. While many patients achieved clinically meaningful reductions in proptosis by week 12, ~1/3 did not. Here we show that this subgroup of patients continued to show improvements in proptosis and diplopia as treatment progressed from 12 to 24 weeks. A substantial proportion of these patients went on to achieve a clinically meaningful improvement in proptosis (the main study outcome) at 24 weeks. In this group of patients, age, sex, or duration of GD did not have an impact on differences between response at 12 and 24 weeks. The proportion of diplopia responders also increased between weeks 12 and 24, as did the proportion of patients who achieved disease inactivation (CAS 0/1). In contrast, almost all patients in the subgroup had experienced clinically significant improvements in CAS by week 12. Similar improvements in proptosis and diplopia were not seen in patients who received placebo. The results of this study indicate a temporal variation in the response to teprotumumab therapy.

The heterogenous response to teprotumumab therapy at 12 weeks likely reflects the diversity in pathways downstream of the IGF-1R. This pathway is a complex and tightly regulated network [19]. There are six serum insulin-like growth factor binding proteins, which serve as regulators of the pathway by determining ligand bioavailability [20]. Binding of IGF-1/2 to IGF-R occurs as a homodimer or heterodimer with insulin receptor A or B (IR-A and IR-B) [21]. There are six different IGF-IR/IR combinations that might occur, and formation of these hybrids appears to be random, with certain combinations displaying a greater affinity for IGF-1 than others [22]. Furthermore, the IGF-IR can heterodimerize with members of other receptor families, for example, epidermal growth factor [23]. These potential combinations substantially enlarge the repertoire of signaling events, which might occur along the IGF-1R pathway and might explain the heterogeneity of response to teprotumumab therapy.

Although the study was retrospective, the data for the patients included in this study were obtained from two randomized, placebo-controlled studies with strict inclusion and exclusion criteria.

This work suggests there is variability in the time taken to manifest a clinically significant response to teprotumumab therapy, and some patients may need a longer time to respond. This should encourage clinicians to allow patients to complete a full 24-week course of teprotumumab, even if only modest results are seen during the initial stages of therapy.

## Summary

### What was known before

- Teprotumumab reduces proptosis and signs of inflammation.

### What this study adds

- There is variability in time to clinical response (>2-mm-proptosis reduction).
- Of those patients who did not have a significant reduction in proptosis at 12 weeks, 63% went on to have a significant change.
